# Melioidosis in Malaysia: Incidence, Clinical Challenges, and Advances in Understanding Pathogenesis

**DOI:** 10.3390/tropicalmed3010025

**Published:** 2018-02-27

**Authors:** Sheila Nathan, Sylvia Chieng, Paul Vijay Kingsley, Anand Mohan, Yuwana Podin, Mong-How Ooi, Vanitha Mariappan, Kumutha Malar Vellasamy, Jamuna Vadivelu, Sylvia Daim, Soon-Hin How

**Affiliations:** 1 School of Biosciences and Biotechnology, Faculty of Science and Technology, Universiti Kebangsaan Malaysia, Bangi 43600, Malaysia; sylvia@ukm.edu.my; 2 Emergency Department, Pantai Hospital Ipoh, 31400 Ipoh, Malaysia; paulkvijay@gmail.com; 3 Department of Paediatrics, Bintulu Hospital, Bintulu 97000, Malaysia; anand_bintulu@yahoo.com; 4 Institute of Health and Community Medicine, Universiti Malaysia Sarawak, Kota Samarahan 94300, Malaysia; pyuwana@unimas.my (Y.P.); monghowooi@gmail.com (M.-H.O.); 5 Department of Paediatrics, Sarawak General Hospital, Kuching 93586, Malaysia; 6 Department of Medical Microbiology, Faculty of Medicine, University of Malaya, Kuala Lumpur 50603, Malaysia; vanitha.ma@gmail.com (V.M.); kumuthamalar@um.edu.my (K.M.V.); jamuna@ummc.edu.my (J.V.); 7 Department of Pathobiology and Medical Diagnostics, Faculty of Medicine and Health Science, Universiti Malaysia Sabah, Kota Kinabalu 88400, Malaysia; sylviadaim@ums.edu.my; 8 Department of Internal Medicine, Kulliyyah of Medicine, International Islamic University Malaysia, Kuantan 25200, Malaysia

**Keywords:** melioidosis, *Burkholderia pseudomallei*, Malaysia, epidemiology, bacteriology

## Abstract

Malaysia is an endemic hot spot for melioidosis; however, a comprehensive picture of the burden of disease, clinical presentations, and challenges faced in diagnosis and treatment of melioidosis is not available. This review provides a nonexhaustive overview of epidemiological data, clinical studies, risk factors, and mortality rates from available literature and case reports. Clinical patterns of melioidosis are generally consistent with those from South and Southeast Asia in terms of common primary presentations with diabetes as a major risk factor. Early diagnosis and appropriate management of Malaysian patients is a key limiting factor, which needs to be addressed to reduce serious complications and high mortality and recurrence rates. Promoting awareness among the local healthcare personnel is crucial to improving diagnostics and early treatment, as well as educating the Malaysian public on disease symptoms and risk factors. A further matter of urgency is the need to make this a notifiable disease and the establishment of a national melioidosis registry. We also highlight local studies on the causative agent, *Burkholderia pseudomallei*, with regards to bacteriology and identification of virulence factors as well as findings from host–pathogen interaction studies. Collectively, these studies have uncovered new correlations and insights for further understanding of the disease.

## 1. Introduction

### 1.1. Historical Background

**Figure 1:**
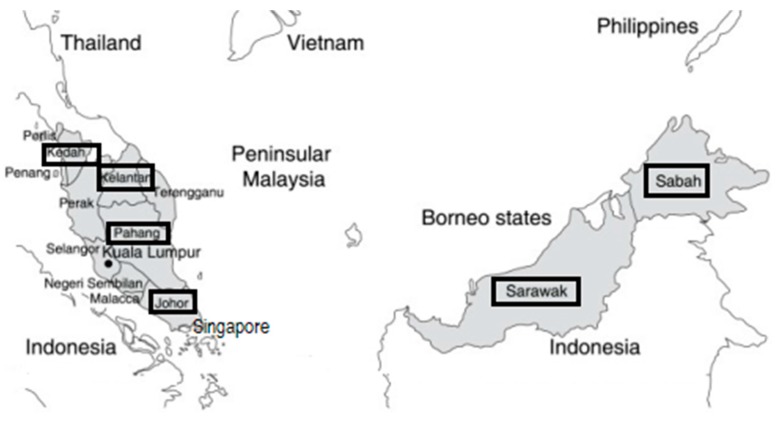
A map of Malaysia indicating the major states with reported cases of melioidosis (black boxes) presented in this review. The majority of case reports are from hospitals and medical centers in Pahang and Sabah due to the diligence of the state health authorities in initiating state-level registries for melioidosis.

Melioidosis was first documented in what was then known as Malaya in an outbreak in 1913 involving laboratory guinea pigs and rabbits at the Institute for Medical Research, Kuala Lumpur [1]. Since then, melioidosis has been recognized as an endemic disease in Malaysia. Stanton et al. also described two of the earliest human cases in Kuala Lumpur who presented with symptoms consistent with melioidosis and whose autopsy swabs grew *Bacillus whitmori* [2]. In 1932, Stanton and Fletcher described a total of 39 cases of melioidosis in Kuala Lumpur [3]. There was a long hiatus in documented melioidosis cases in the Malayan Peninsula, possibly due to World War II and post-World War II insurgencies that plagued the Indo-China region through the 1950s and 1960s. In 1957, Malaya gained independence from the United Kingdom and the Malaysian Federation was subsequently formed with the union of Malaya, Singapore, Sabah, and Sarawak in 1963. With the stabilized geopolitical climate, interest in melioidosis was reignited with environmental surveillance and serosurveillance conducted in several states by the United States of America Medical Research Unit (USAMRU) in the 1960s [4,5,6,7]. In the nationwide serosurveillance study, participants from Kedah and Sabah (Figure 1) recorded the highest seroprevalence against *B. pseudomallei* [5], while *B. pseudomallei* was isolated from soil and water from several states in Peninsular Malaysia and Sabah [4,6,7]. In 1970, Thin et al. described ten cases of melioidosis involving military personnel presenting at military hospitals in Malacca (Melaka) and Singapore over a three-year period [3].

Cases of animal melioidosis are not rare, nor are they unfamiliar to veterinarians in Malaysia. Recorded reports of animal melioidosis date back to almost 100 years ago, beginning with the 1921 seminal work of Stanton and Fletcher [8]. Since then, melioidosis has been reported in local goats, sheep, cattle, pigs, deer, monkeys, horses, cats, dogs, and rabbits [9,10,11,12,13]. In addition, a number of State Veterinary Services Departments in Malaysia keep records of animal melioidosis cases for surveillance and control purposes, mainly as a means to mitigate loss of animals of economic importance, especially ruminant livestock. Fatal melioidosis was also reported among orangutans at the Sepilok Rehabilitation Centre in Sandakan, Sabah in 1965 and 1968, where *B. pseudomallei* was successfully isolated from the Centre [14]. Primates are indeed susceptible to *B. pseudomallei* infection, as reported in Johor and Kuala Lumpur in the 1960s, involving pig-tailed macaques, a spider monkey, and a gibbon [10]. The variety of animals reported to be affected by melioidosis and the many locations from which the animal cases came, suggest a wide distribution of *B. pseudomallei* in the environment in Malaysia. Although direct zoonotic transmission of melioidosis appears rare in the country, with only one published report available thus far of a case of transmission from sheep to human [15], the risk may be underestimated. Furthermore, the risk of indirect zoonotic transmission, which could potentially occur via environment contaminated with *B. pseudomallei* shed from infected animals, has not been properly assessed. For the better management and control of human melioidosis in the country, cases of animal melioidosis should not be taken lightly.

Research led by Malaysian clinicians and microbiologists on melioidosis started to gain momentum in the late 1980s and early 1990s with numerous publications—in particular, clinical reports and reviews [16,17,18,19]. The research interest subsequently expanded to include molecular microbiology, genomics, and pathogenesis [20,21,22,23,24,25,26,27,28], with the availability of better facilities, funding, and trained local experts.

### 1.2. Modes of Transmission

The *B. pseudomallei* natural environmental habitat in endemic areas is soil and water. Most cases of melioidosis occur in persons with regular contact with contaminated soil or water, via penetrating wounds or pre-existing skin abrasions [29]. Inhalation via contaminated dust or water as in severe wet weather conditions is the next most common route of entry and is characterized by pneumonia and more severe infection [30]. Heavy rains precipitate flooding, which facilitates churning of *B. pseudomallei* to the surface soil, aerosolizing the bacteria and increasing exposure potential. Zueter et al. [31] in their case series from Kelantan observed that the highest frequency of admissions occurred during the rainy season from November to February. Hassan et al. [32] found that cases and deaths from melioidosis in the Alor Setar region of Kedah increased linearly with mean monthly rainfall. Pagalavan [33] reported a case after a near-drowning episode, where the patient most probably acquired the infection through aspiration.

## 2. Burden of Disease and Epidemiology

Melioidosis is not a notifiable disease in Malaysia; therefore, the true incidence of melioidosis in Malaysia is unknown, although more than a thousand cases have been reported throughout Malaysia [34]. Incidence may vary between states, and even within the same state, there may be various hotspots [35]. States that are active in agriculture generally report a higher incidence of melioidosis. Pahang, the largest state in Peninsular Malaysia, where agriculture is the main economic activity, recorded incidences of culture-confirmed adult melioidosis of 6.1 per 100,000 population per year from 2000–2003 [36]. The state of Kedah, which is situated at the Malaysia–Thailand border and is the largest rice producer in Malaysia, reported an incidence of 16.35 per 100,000 population a year [32]. Table 1, Table 2 and Table 4 present selected results from six studies pertaining to Malaysia. Four of these studies used data from systematic compilation of cases from hospital laboratories [18,31,33,36], the fifth used data from a registry [32], and the sixth is a synthesis of published case reports from Malaysia providing more detailed information on individual cases [37]. Table 3 presents results from four studies on paediatric melioidosis in Malaysia.

### 2.1. Demography and Risk Factors

Melioidosis may occur at any age, including newborns. The peak incidence in the Malaysian case series is between 40 and 60 years of age (median, 44–51 years) (Table 1), the age range during which most co-morbid conditions develop. A preponderance of the disease among males was noted; the gender difference may be due to a higher potential for males to be involved in soil-related occupations and activities facilitating exposure. In four of five case series, most cases were reported in persons of Malay ethnicity, possibly reflecting the higher proportion of Malay rice paddy farmers and agriculture employees with potential for exposure to contaminated water and/or soil [36] or the predominant Malay ethnic composition of certain states [31,32,33].

Most cases (58–85%) had at least one risk factor reiterating *B. pseudomallei*’s classification as an opportunistic pathogen and that susceptibility of the host is a vital factor in the acquisition of infection. More than one risk factor was reported in 8.1–36% of cases. No risk factor was reported in 15–42% of cases, possibly reflecting under-reporting, unknown residual factors, and high bacterial load or inhalation route in some cases (Table 1). Data on environmental risk factors was sparse in all of the Malaysian case series, underscoring the need to acquire this important information to assist in the education and prevention of melioidosis. Workers in the agricultural and construction sectors, military personnel, eco-tourists, and persons involved in rescue operations are groups known to be at high risk because of their contact with contaminated soil or water [38,39,40]. Employment in the farming/fishing/forestry industry was reported in 2–25% of cases followed by 3–18% for the construction/trucking industry. Four cases were involved in rescue operations in the recreational forests of Pahang; all of the four cases had co-infection with leptospirosis and three of the four had a fatal outcome [37,40].

Several underlying medical conditions or drug therapy that may impair host defense predispose individuals to melioidosis [41,42]. As reported in other endemic areas of the world, type 2 diabetes mellitus is the most common co-morbid condition associated with melioidosis in Malaysia; 38–75% of melioidosis patients were either newly diagnosed or had pre-existing type 2 diabetes mellitus (Table 1). Other co-morbid conditions associated with melioidosis include chronic renal disease (6–19%), tuberculosis (9–16%), immune disorders/steroid therapy (2.9–9.5%), solid tumors (0.7–10%), haematological malignancies (0.7–8%), chronic lung disease (2.8–3.0%), chronic heart disease (7.0%), smoking (10%), chronic alcoholism (0.7–2.0%), hemolytic anaemia (0.7–2.0%), and malnutrition/anaemia (8%).

### 2.2. Clinical Presentation

Melioidosis presents as a febrile illness with protean clinical manifestations, ranging from acute fulminant pneumonia and/or septicemia mimicking other community-acquired infections, to a chronic infection that may mimic tuberculosis or malignancy. The disease is characterized by abscess formation in multiple organs and is referred to as ‘the great mimicker’ because of its similarity to other infections that obscure its correct diagnosis [38]. The pitfalls and optimal approaches to diagnosis have been previously reviewed by Kingsley et al. [43] and are highlighted in a latter section.

In the Malaysian case series, more than 90% of cases were of acute onset [31,37], presenting as acute respiratory infection, acute bacteraemia, or soft tissue infection with fever almost always present. Soft tissue infections include infections of nonskeletal tissue surrounding or supporting organs and other structures including subcutaneous tissue, muscle, lymph nodes, blood vessels, and soft tissue organs (namely, the liver or spleen). Less than 10% of cases were chronic in onset (symptoms more than two months), presenting as chronic pneumonia, chronic skin ulcers/abscesses, and disseminated infection progressing to sepsis, while subclinical infections have also been documented. The major reasons for emergency hospital admissions were acute pulmonary infection progressing to acute respiratory failure, acute bacteraemia progressing to septic shock, severe soft tissue infection, or pyrexia of unknown origin [31,37,43].

The clinical spectrum of melioidosis may be classified into four non-mutually exclusive categories (Table 2): (1) Acute pulmonary infection presenting as pneumonia is the most common clinical presentation, reported in 33–63% of the Malaysian case series. The infection may be primarily acquired via inhalation or alternatively via hematogenous spread following inoculation. (2) Acute blood stream infection was reported in 19–61% of Malaysian case series; patients with co-morbid disease such as diabetes are more likely to present with this form of the disease. Patients may present with a history of fever (median, 6 days; range, 3 days–several months), respiratory distress, abdominal discomfort, muscle tenderness, and disorientation. The clinical picture may vary from a simple bacteraemia with no evident focus of infection, to fulminant septic shock and multiorgan abscesses with 16–34% of cases presenting with septic shock. (3) Disseminated infection, occurring in 16–37% of cases, presents with symptoms of fever, weight loss, abdominal pain, muscle and joint pain, headache, and seizures, and with clinical signs of abscess formation in multiple organs with or without bacteraemia. (4) Acute localized infection, occurring in about 10% of cases, may present as skin ulcers, subcutaneous tissue abscesses, parotid abscess, or ocular infection. The infection may remain localized or may rapidly progress through the blood stream to more widespread infection.

The lungs were the most common site of primary infection followed by soft tissue and bone/joint infection (Table 2). Genitourinary and neurological infections were less frequent. Mortality occurring within 24–48 h of admission precluded a complete workup to identify the site of infection. Pulmonary melioidosis (41–58%) has been observed for both acute and chronic forms of lung involvement. The chronic form of lung involvement mimics tuberculosis where patients present with symptoms of fever and cough with purulent sputum and about one-third of patients possibly having haemoptysis. Pleural involvement occurred in 9–33% of cases and thoracic empyema was occasionally seen. Soft tissue and skin infection (17–36%) is the second most common primary site of presentation. Soft tissue involvement manifests as subcutaneous, intramuscular, and deep-seated abscesses with no particular preference for specific anatomical sites. *B. pseudomallei* could occasionally be isolated from aspiration of abscesses/skin pustules or skin biopsies. Bone and joint infections (6.0–13%) present mainly as septic arthritis, most commonly affecting the knee, followed by the ankle, wrist, and elbow joints, whilst osteomyelitis was less common. Genitourinary infections (3.2–10%) presented as prostatic abscesses, pyelonephritis, perinephric abscesses, or scrotal abscesses. Meanwhile, neurologic melioidosis (4.8–7.5%) presented mainly as pyemic such as brain abscesses, subdural empyema, epidural abscesses, etc. [37]. Hassan et al. [32] and Puthucheary et al. [18] also reported only brain abscesses in their case series whilst meningoencephalitis was uncommon. A similar pattern of pyemic lesions of neurological melioidosis was noted among three cases reported from a registry in Sarawak [44]. The clinical scenario of no identified focus of infection (7.5–24%) was more likely to occur in bacteraemic than nonbacteraemic patients [37].

Besides the primary infection, it was not uncommon for secondary foci of infection to occur. Overall, 49% of cases had secondary foci of infection. Secondary subcutaneous tissue abscesses (21%) were most common in all primary diagnostic groups followed by secondary pneumonia [37]. Liver (4.0–18%) and splenic (2.0–12%) abscesses were commonly found in bacteraemic cases (Table 2) with the ‘honeycomb’ or ‘Swiss cheese’ appearance of liver and spleen which is characteristic of melioidosis. Case reports noted a frequency of 13% of prostate abscesses in male patients [37], whereas the case series reported a lower proportion (0.9–2.6%) although this could be attributed to the more complete investigational workup available for case reports than case series. Other rare presentations of melioidosis reported in Malaysia include mycotic pseudoaneurysm (7.5%), pericardial effusion (1.0–2.0%), and heart valve vegetation (3.0%). In mycotic pseudoaneurysm, a pulsatile abdominal mass was the predominant clinical sign and fever was a consistent clinical feature. Diagnosis was made on the basis of CT findings; aneurysms were located in the major abdominal arteries and most had surgical intervention [37].

In summary, the clinical patterns of cases reported from Malaysia are consistent for the most part with previous case reports from South and Southeast Asia where pneumonia is the most common primary presentation followed by soft tissue abscesses with diabetes a major risk factor [37]. Concomitantly, symptoms more frequently observed in Malaysian patients included primary neurological infection and internal foci of infection such as abscess of the liver, spleen, and prostate, and mycotic pseudoaneurysms were higher than previously reported in the region [37]. Neurological melioidosis is primarily pyemic in Malaysia; the distinct syndrome of brain stem encephalitis with flaccid paralysis noted in 4% of melioidosis cases in northern Australia [30] was uncommon in Malaysia and was reported in less than 1% of cases [37].

### 2.3. Paediatric Melioidosis in Malaysia

*B. pseudomallei* infections are reported less commonly in children than in adults [45]. In Malaysia, paediatric melioidosis has been described in detail in only four studies, reporting between 13 and 42 culture-confirmed cases each (Table 3). These studies, describing children with melioidosis from both Peninsular Malaysia (Pahang [46], Kuala Lumpur [47]) and Malaysian Borneo (Sabah [44], Sarawak [35]), have shown varying incidences and host risk factors for disease. However, all report high rates of disseminated disease and septicaemia with high case fatality rates. The incidence of paediatric melioidosis has been estimated to be 0.6, 0.7, and 4.1 per 100,000 children in Sabah, Pahang, and Sarawak, respectively. Marked regional variations in incidences are known, although the reasons for these variations remain unclear. For example, incidences as high as 20.2 per 100,000 children were reported in some districts in Sarawak, while no cases were documented in other districts [35].

The importance of an underlying medical condition in the predisposition to childhood melioidosis varied between the different studies in Malaysia. In the study from Kuala Lumpur, 69% of children had an underlying medical condition, mainly haematological malignancy [47]. In Sabah, where high rates of β-globin gene deletions are found in the local ethnic population, 41% of children had thalassemia major. Interestingly, this higher incidence of melioidosis among children with transfusion-dependent thalassemia reduced significantly with the institution of iron chelation therapy, indicating that it was the iron overload that was important in the pathogenesis [44]. Other conditions documented in these studies included primary immunodeficiency, renal failure, diabetes mellitus, hypoaldosteronism, albinism, congenital heart disease, and malnutrition. In contrast, no underlying medical conditions were noted in children with melioidosis in Pahang and Sarawak. However, 32% of those in Sarawak were noted to have poor nutritional status and this may also be an important host risk factor.

In contrast to the paediatric melioidosis literature from most endemic regions [48,49], a large proportion of children with melioidosis in Malaysia presented with disseminated or septicaemic disease. This proportion ranged between 44% and 93% in the various studies. Pneumonia was the predominant manifestation in those with disseminated disease, occurring in as many as 76–83% of cases in Sabah [44] and Sarawak [35]. Undifferentiated fever, with no overt focus of infection, was another important manifestation, occurring in over 28% of children with disseminated melioidosis in two of the studies. Septic arthritis and osteomyelitis occurred in almost 10–15% of children in the Bornean studies. Splenic (and, less frequently, liver) abscesses were also common findings; splenic abscesses were noted in >50% of children in Sabah and Sarawak who had abdominal ultrasound imaging. Neurological involvement was less common, documented in a total of nine children overall. As has been reported in other melioidosis endemic regions, neonatal infections also occurred [50].

Localized melioidosis infection in Malaysian children typically involved either lymph nodes (mainly cervical), skin and soft tissue, or the lacrimal glands. Parotid infections were rare, documented in only 3% of children, in stark contrast to the >25% of children who present with this manifestation in Cambodia and Thailand [49,51]. Children with melioidosis in Malaysia had extremely high fatality rates. Overall, between 24–59% of culture-confirmed melioidosis cases had a fatal outcome. Bacteraemia, disseminated disease, and involvement or dysfunction of a high number of organs were associated with poorer outcomes. Case fatality rates invariably exceeded 75% when septicaemic shock was present. In addition, those with underlying medical conditions had higher fatality rates. In contrast, children with localized disease had significantly better outcomes with only one death recorded.

A delay in diagnosis and in initiation of appropriate antimicrobial treatment was observed in most paediatric melioidosis studies in Malaysia. In Sarawak, the diagnosis was initially missed by nearly 90% of primary healthcare providers and the median duration of symptoms was 14 days before these children were finally admitted to hospital. In Sabah, an appropriate antimicrobial was initiated at admission in <50% of children. These delays likely contributed to the high fatality rates observed and highlight the lack of awareness both in the community and in healthcare professionals in Malaysia.

## 3. Laboratory Diagnosis of Melioidosis in the Malaysian Healthcare System

Melioidosis is a challenging infectious disease to diagnose even for an endemic country like Malaysia. The absence of pathognomonic clinical presentations, coupled with the lack of familiarity with the disease among attending physicians and laboratory personnel, are the main factors contributing to misdiagnoses especially in rural settings. In Malaysian public tertiary hospitals equipped with modern-day microbiology facilities, laboratory diagnosis of melioidosis is typically included in the routine blood culture test that is done as part of the sepsis workup for patients with fever. Although Ashdown’s agar is widely used in other melioidosis-endemic countries [52,53], it is not widely used in diagnostic laboratories in Malaysia. Instead, Francis media agar [21], MacConkey agar, blood agar, and chocolate agar are the common media used in public healthcare, where use of different combinations of agar media varies from one hospital to another.

Once isolated from clinical samples, confirmation of *B. pseudomallei* is either by manual (API 20NE biochemical kit, bioMérieux, Marcy-l’Étoile, France) or automated biochemical systems (Vitek 2, bioMérieux, France; BD Phoenix, Becton Dickinson, Franklin Lakes, NJ, USA; MALDI-TOF MS, Bruker, Bremen, Germany) for any bacterial isolates initially confirmed to be nonmotile, Gram-negative, and oxidase-positive bacilli. Nonetheless, there are also pitfalls in biochemical tests where *B. pseudomallei* isolates were misidentified as other *Burkholderia* species, as previously observed by Podin et al. [54]. Two tests recommended in the guidelines of the Melioidosis Diagnostic Workshop 2013 [55] are not currently included in the routine clinical microbiology laboratory identification workflow, due to various reasons such as budgetary constraints and regional variations of *B. pseudomallei* phenotypes: antibiotic susceptibility tests for amoxicillin-clavulanate, colistin, and gentamicin, and the *B. pseudomallei*-specific latex agglutination assay. While highly sensitive, rapid, and specific, the use of *B. pseudomallei*-specific latex agglutination assay is too costly for public hospitals. Although antibiotic susceptibility tests for amoxicillin-clavulanate, colistin, and gentamicin are not done routinely, this diagnostic algorithm is certainly worth adopting in Malaysia as it has been shown to be useful in resource-limited laboratories in north-central Vietnam [56]. However, gentamicin-susceptible isolates need to be assessed with extra care due to the discovery of gentamicin-susceptible strains that are predominantly found in Central Sarawak [26]. On average, about 3–5 days are still needed for most hospital laboratories in Malaysia to diagnose melioidosis: 20–48 h of blood and agar plate culture, followed by 48–72 h of biochemical identification processes.

Serological tests to detect the presence of anti-*B. pseudomallei* antibody titers using either the indirect hemagglutination assay (IHA) or enzyme-linked immunosorbent assay (ELISA) are widely accepted as unreliable for the diagnosis of melioidosis in Malaysia. The current use of IHA and ELISA in the country is limited to contact investigations and interim monitoring of persons found associated with melioidosis-confirmed cases of either humans or animals. Mohd Noor et al. [57] reported the potential application of their optimized in-house IgM ELISA method for diagnosis of acute melioidosis. The robustness of this assay in diagnosing acute melioidosis in public hospital settings remains to be seen and is pending validation. At the institutional level, the Institute for Medical Research, which is the biomedical research arm of the Malaysian Ministry of Health, has since replaced IHA with the optimized in-house IgM ELISA as the method of choice to test for recent exposure to *B. pseudomallei*. Although not standardized at the national level, molecular methods for confirmation of *B. pseudomallei* are also available and doable in Malaysia using either conventional polymerase chain reaction (PCR) or real-time PCR on the type three secretion system (TTSS)-1 and other gene targets with high levels of specificity and sensitivity [58]. If carefully adapted into the existing melioidosis diagnostic workup, these molecular methods may have a shorter diagnostic turnaround time. Nonetheless, bacterial culture cannot be totally abandoned as direct detection of *B. pseudomallei* remains complicated due to presence of inhibitors [59].

## 4. Mortality and Recurrence

Based on incidence and mortality of melioidosis in Malaysia, it is estimated that more than 2000 patients die of melioidosis per year, which is much higher than death resulting from dengue or tuberculosis infection. Despite advances in treatment, the case fatality ranged from one-third to about half of patients (33–54%) in four of the five Malaysian case series and in the review of case reports that included all cases, irrespective of bacteraemic status (Table 3). However, when stratified by bacteraemic status, the mortality was about threefold higher among bacteraemic cases compared with nonbacteraemic cases (48–65% vs. 19%). This was previously observed by Puthucheary et al. [18] when they reported 65% mortality, selected on the basis of positive bacteraemic status. A study evaluating patients with bacteraemic melioidosis in Kelantan also reported up to 63% mortality [60]. The normal pathological consequence of bacteraemia is septic shock. Septic shock was the strongest predictor for mortality; in most cases, signs of septic shock occurred within 24 h of admission, presenting as acute respiratory distress syndrome. The mortality among cases with septic shock was 100% compared to 30% among cases without septic shock [37]. Zueter et al. [31] also concluded that septic shock [odds ratio (OR) = 16.5, 95% confidence interval (CI = 6.1–44.9)] was the strongest predicting factor for mortality adjusted for other factors.

With regards to other factors contributing to mortality, Zueter et al. [31] found that age >40 years (OR = 6.47, 95% CI = 1.7–23.8) and the presence of at least one co-morbid condition (OR = 3.0, 95% CI = 1.1–8.4) were independent predicting factors. Among co-morbid conditions, diabetes mellitus was the major underlying risk factor for mortality; 69% of patients had diabetes mellitus in the case series by Hassan et al. [32]. With regards to organ involvement, pneumonia and bacteraemia accounted for most deaths. How et al. [36] found that patients with pneumonia, multiple organ involvement, and bacteraemia had a statistically-significant higher mortality than patients with subcutaneous, musculoskeletal, or internal organ involvement without pneumonia; mortality from acute pneumonia was about 65–73% [18,32,36,37]. Hassan et al. [32] reported that patients with soft tissue abscesses were also at risk for mortality and that osteomyelitis/septic arthritis and liver and splenic abscesses were good predictors of mortality among bacteraemic cases. Patients with pneumonia had approximately threefold higher mortality than those with soft tissue abscesses (63% vs. 18%, *p* = 0.003) [37]. Zueter et al. [31] found that 23% of fatal melioidosis cases were directly attributable to lack of prompt acute-phase treatment.

Patients who survive an initial episode of acute melioidosis have a high potential to develop clinical recurrence, possibly due to failure of the host to eliminate the organism during the initial episode of infection (relapse) or due to reinfection. In Malaysia, the reported rate of culture-confirmed clinical recurrence varied from 2.6% to 19% (Table 4). Chaowagul et al. [61] reported a twofold higher recurrence rate of 15–30% per year in northeast Thailand. The lower recurrent infection rate in Malaysia is perhaps an underestimate, reflecting the high proportion of cases lost to follow-up and shorter duration of follow-up. Published data on the recurrence rate in Malaysia did not specifically note the proportion of relapse and reinfection because serotyping is not routinely performed for clinical isolates.

Similar to tuberculosis, melioidosis infection may be dormant with prolonged latency [11]. Factors contributing to dormancy include survival of *B. pseudomallei* in protected environments, such as phagocytic cells or enclosed abscesses, or the ability of the organism to form a protective covering in infected tissues where antimicrobials cannot penetrate [11]. Reactivation from a latent focus and recurrence into a fulminating form may occur when host defense is compromised as in diabetes mellitus [62]. Risk factors for recurrence amongst Malaysian patients included severity of disease (positive blood culture, multifocal disease), incomplete or inadequate treatment with amoxicillin-clavulanate during the intensive phase of treatment, and improper eradication therapy—amoxicillin-clavulanate, oral quinolones, or doxycycline monotherapy—and nonadherence or duration less than 12 weeks [11]. The most important factor predisposing to relapse is nonadherence to eradication therapy (oral antimicrobial therapy) or inadequate antibiotic therapy. Recurrence was noted to occur in immunocompromised patients despite the full course of microbial therapy. Zueter et al. [31] reported that incomplete treatment, or missed or delayed diagnosis, contributed to the occurrence of recurrent infection among four patients in their case series, all of whom died during the recurring episodes. How et al. [36] reported that patients who did not receive specific therapy and those who received specific therapy for less than two weeks had a 40% and 25% higher risk of relapse, respectively.

## 5. Molecular Pathogenesis of *B. pseudomallei*

### 5.1. B. Pseudomallei Bacteriology

*B. pseudomallei* is intrinsically resistant to a diverse group of antibiotics including penicillins, rifamycins, aminoglycosides, and many third-generation cephalosporins. It is also relatively resistant to quinolones and macrolides, limiting options for therapeutic treatment of melioidosis [11]. Among 81 *B. pseudomallei* isolates from Malaysia tested against nine different antimicrobial agents, susceptibility to ceftazidime, amoxicillin-clavulanic acid, meropenem, imipenem, and trimethroprim-sulfamethoxazole was noted [63]. Despite the high percentage of susceptibility reported, it was interesting to note that the overall results highlighted the emergence of multidrug-resistant isolates. Of the 81 isolates, six were found to carry *bpeB*, *amrB*, *penA*, and BPSS1119 genes, believed to be associated with multidrug resistance. Although the majority of *B. pseudomallei* in Malaysia is gentamicin resistant, more than 80% of *B. pseudomallei* from Central Sarawak of Malaysian Borneo were found to be susceptible to aminoglycosides and macrolides, attributed to a novel nonsynonymous mutation within the *amrB* gene of the AmrAB-OprA efflux pump [26]. Unaltered virulence was observed for these gentamicin-sensitive isolates, suggesting that the loss of aminoglycoside and macrolide resistance has little consequence for virulence and might even enhance environmental survival of these isolates. Thus far, this intriguing phenomenon has not been observed in other parts of Malaysia. A recent study by Zueter et al. [64] on genotyping of 83 clinical *B. pseudomallei* isolates from Peninsular Malaysia revealed 32 different sequence types (STs), of which 13 were novel. All non-novel STs were previously identified in other Asian countries [65,66], suggesting that Malaysian isolates may not be distinct from those of Southeast Asian countries. A lack of relationship between *B. pseudomallei* STs and clinical melioidosis presentation agrees with previous studies indicating an absence of association between any ST and disease outcome, but host and environmental factors are possible reasons for the diverse nature of the clinical presentation of melioidosis [67,68].

### 5.2. Host–B. pseudomallei Interaction and Identification of Potential Virulence Factors

Host–pathogen interaction studies on *B. pseudomallei* have been actively undertaken in Malaysia, facilitated by the availability of various established host model systems, such as in vitro cell-based models and in vivo models (vertebrate and invertebrate). Findings from these studies have contributed new knowledge to the field of melioidosis pathogenesis and the identification of new potential virulence factors as well as mechanisms of immune response subversion.

Chin et al. [69] investigated the host transcriptional response in a murine acute-phase melioidosis model through microarray-based expression profiling, and highlighted the vital link between innate and adaptive immunity during *B. pseudomallei* infection. They demonstrated that TLR2 was induced to initiate an inflammatory response, followed by an increase in transcripts associated with cell death, caspase activation, and peptidoglysis that ultimately promote tissue injury in the host [69]. In addition, suboptimal activation and function of the downstream complement system correlated with uncontrolled spread of bacteria, eventually leading to death of the infected host. In a parallel study on a diabetic model of acute melioidosis, Chin et al. [70] suggested that the presence of elevated glucose levels impaired the host innate immune system by delaying the identification and recognition of *B. pseudomallei* surface structures. Subsequently, this resulted in delayed activation of various inflammatory and immune responses, as well as the general ‘alarm signal’ of infection, which may contribute to the increased susceptibility of individuals with pre-existing diabetes to melioidosis [70].

Utilizing the invertebrate model *Caenorhabditis elegans*, the team at Universiti Kebangsaan Malaysia demonstrated that direct prolonged interaction between *C. elegans* and *B. pseudomallei* is required for a complete lethal effect, suggesting that live or proliferating bacteria continuously produce toxins in order to mediate the full killing effect [71,72]. To explore the possibility of toxin-mediated killing, Ooi et al. [72] demonstrated over-expression of the *C. elegans* ABC transporter gene, *pgp-5*, upon *B. pseudomallei* infection, suggesting that the host actively thwarted the pathogenic assaults during infection. A genome-wide transcriptome analysis of infected *C. elegans* revealed a previously-undescribed mechanism by which *B. pseudomallei* suppressed host immunity by specifically targeting an intestinal transcription factor, GATA/ELT-2, thus reducing its availability and consequently inhibiting the expression of GATA transcriptional targets, which include host defense effectors [73].

Chieng et al. [74] conducted a study to understand *B. pseudomallei* adaptation to the intracellular environment of macrophage cells and demonstrated that the bacterium adapted rapidly within macrophages through regulation of its metabolism and growth rates. Of note, the type VI secretion system was induced throughout the infection, highlighting its major role in ensuring pathogen survival and replication in the cell cytosol. However, expression of many known virulence factors was suppressed, suggesting possible host immune system avoidance by intracellular *B. pseudomallei* [74]. In a separate study by the group from University Malaya, Vellasamy et al. [27] investigated the host immune response to *B. pseudomallei* infection in lung epithelial cells. They demonstrated the over-expression of several host carbohydrate metabolic pathways and suppression of the alternate complement, coagulation, lysosome, and phagosome pathways, suggesting bacterial adaptation and evasion of the host innate immune response. Overall, new knowledge from host–pathogen interaction studies of *B. pseudomallei* has revealed mechanisms by which the host responds and some of the mechanisms by which the pathogens avoid host defenses, thereby surviving and growing in host cells. This information should contribute to the identification of new therapeutic targets and vaccine candidates.

The ability of *B. pseudomallei* to adhere, invade, survive, and replicate within mammalian host cells is among the key factors in its pathogenesis [28]. A cohort of Malaysian human, animal, and environmental *B. pseudomallei* isolates was characterized by various biochemical assays to determine the secretion of selected virulence determinants [75,76]. A proteome analysis of *B. pseudomallei* culture supernatant identified metabolic enzymes, transcription/translation regulators, potential virulence factors, chaperones, transport regulators, and hypothetical proteins, several of which were immunoreactive [77]. This study was extended to further evaluate the role of the cell invasion protein, BipC, in pathogenesis of *B. pseudomallei*. BipC, an immunoreactive protein, is involved in actin binding to facilitate internalization of *B. pseudomallei* into host cells, as a bipC mutant was impaired in adherence, invasion, and intracellular survival in epithelial cells, and BipC protein is required for full virulence in a murine model of melioidosis [78,79]. Recently, Vadivelu et al. [80] showed that *B. pseudomallei* localized within the nuclear compartment of host cells, suggesting that the nucleus may play a role as an occult or transient niche for persistence of intracellular pathogens, potentially leading to recurrent episodes or recrudescence of infection.

*B. pseudomallei* is also known to form biofilm, an important aspect in bacterial pathogenesis due to its ability to promote bacterial survival or spread within the host and protection from antibiotics [81]. Small colony variants (SCVs) of *B. pseudomallei,* which displayed significantly greater capacity to form biofilms, were shown to be less lethal in a *C. elegans* infection model compared to the K96243 isolate, reflecting theSCV ability to persist in the infected host. Recently, Chin et al. [82] noted that genes involved in surface-associated motility, surface composition, and cell wall biogenesis were over-expressed in a high biofilm producer and are probably required for the initial attachment of biofilms. Up-regulation of genes related to the two component signal transduction systems and a denitrification enzyme pathway suggest that the *B. pseudomallei* high biofilm producer is able to sense the surrounding environmental conditions and regulate the production of extracellular polymeric substance matrix, a hallmark of microbial biofilm formation [82].

Overall, in vivo and in vitro studies using experimental melioidosis animal and cell culture models have aided in revealing a variety of bacterial factors that may contribute to survival, pathogenicity and long-term persistence of *B. pseudomallei* within the host.

## 6. Challenges and Future Perspectives

A major challenge in the war against melioidosis in Malaysia is the lack of awareness among healthcare personnel and the general public as well as difficulties and limitations of fast and effective diagnosis. Melioidosis molecular diagnostic methods are confined to only a small number of laboratories in research and academic institutions in Malaysia, and are more often employed for research purposes. Two factors have probably prevented the widespread application of molecular methods for routine clinical diagnosis of melioidosis: (1) the seemingly lower demand for these methods compared to those for other infectious diseases such as dengue and tuberculosis, and (2) the unanalyzed cost effectiveness of these molecular methods on melioidosis treatment and management in the country.

Until an effective, portable, and simple diagnostic device is developed for melioidosis, the diagnosis challenge for Malaysia is at least twofold. Awareness about melioidosis among physicians, healthcare personnel, and the general public should be enhanced with periodic and continuous health promotion, education, and/or training. In addition, a diagnostic workflow that is more rapid than the existing one and preferably more robust needs to be developed, validated, and adopted in as many of the public hospitals as possible. In the currently available healthcare services and infrastructure, it is probably more feasible and pragmatic if more people are sufficiently trained to suspect melioidosis, such that patients insist on seeking early medical treatment, while physicians and healthcare personnel are able to initiate empirical treatment and concurrently submit patient samples to the nearest available laboratory for definitive diagnosis in a timely manner. The successful diagnosis of melioidosis, and for many other diseases, requires—at the minimum—the tripartite interaction and cooperation among patients, physicians, and laboratory personnel in Malaysia.

Prevention of infection in areas where the disease is endemic can be difficult since contact with contaminated soil is common. In endemic areas, persons with open skin wounds and those with diabetes or other comorbid conditions should be educated to avoid contact with soil and standing water, as they are at increased risk for acquiring melioidosis. Wearing boots during agricultural work can prevent infection through the feet and lower legs. Post-exposure antimicrobial prophylaxis (PEP) is suggested for at-risk rescue operations workers [83]. In healthcare settings, using standard contact precautions (mask, gloves, gown, and hand washing) is considered sufficient protection.

In some states of Malaysia, the incidence of melioidosis is more frequent, with a large number of people being diagnosed each year. In the state of Pahang, the incidence and mortality rates are relatively high. To tackle this, the Medical Department of the International Islamic University Malaysia (IIUM) with the assistance of the State Health Department, started the Pahang Melioidosis Registry. The aim of this registry is to create awareness among doctors in Pahang on diagnosis and treatment of melioidosis and to reduce patient mortality [84]. In 2014, the Sabah Health Department published a guideline for clinical and public health management of melioidosis in Sabah. The Sabah Melioidosis Registry keeps an account of all cases and local authorities are making efforts to spread awareness about early symptoms and disease management [85].

Melioidosis continues to pose a potential threat, especially in Southeast Asian countries. The relatively low case fatality rate in Malaysia’s neighbor, Singapore, is likely to be related to increased awareness amongst healthcare personnel, resulting in early diagnosis and treatment, optimal antibiotic therapy, and improved supportive management. In Malaysia, there remain many problems in the clinical management of this disease, particularly for patients from rural areas of the country as well as young children. How these issues negatively impact the productivity and socio-economy of the country remains uninvestigated. Low-cost, practical, accurate, and fast detection kits are not available in the market yet. The emergence of intrinsically antibiotic-resistant strains of *B. pseudomallei* and co-infection with leptospirosis are also challenges that have to be addressed quickly. Recently, a network of microbiologists, molecular biologists, and clinicians has been established and is referred to as the Malaysian Melioidosis Network. The aims of the network are primarily (1) to foster cooperation between the bench-scientists and healthcare personnel; (2) to work closely with the Ministry of Health Malaysia and provide informed advice on public awareness, improved diagnostics, and emergence of antimicrobial resistance; and (3) to campaign for melioidosis to be classified as a notifiable disease with well-curated incidence data made available. These efforts are currently ongoing.

## Figures and Tables

**Table 1:** Demographic and risk factors from previously published case series or reports from Malaysia.

	Laboratory or Registry Data					Case Reports
	Zueter et al. [31] (*n* = 158)	Hassan et al. [32] (*n* = 145)	How et al. [36] (*n* = 135)	Pagalavan [33] (*n* = 44)	Puthucheary et al. [18] (*n* = 50)	Kingsley et al. [37] (*n* = 67)
Geographic area	Kubang Kerian, Kelantan	Alor Setar, Kedah	Kuantan, Pahang	Johor Bahru	Kuala Lumpur	Entire country
Data source	1 hospital laboratory	1 hospital-based registry	2 hospital laboratories	1 hospital laboratory	1 hospital laboratory	Published papers
Time period	2001–2015	2005–2008	2000–2003	1999–2003	1976–1991	1975–2015
Inclusion criteria	Confirmed cases	Confirmed cases	Adults (>18 years)	Confirmed cases	Bacteraemia	Confirmed cases
Demographic factors						
Age, median (years)	46 *	50	51	50 *	44 *	44
Male/female ratio	2.8:1	3.0:1	3.6:1	6.3:1	3.2:1	5.1:1
Malay ethnicity %	Most	89	83	71	18	36 ^†^
Risk factors						
Frequency						
At least one %	84	78	85	-	76	58
More than one %	-	-	8.1	-	22	36
None reported %	16	22	15	-	24	42
Environmental exposure				-	-	
Farming/fishing/forestry %	-	19	25	13	2.0	12
Construction/trucking %	-	5.5	-	3.0	18	13
Search/rescue + co-inf. with leptospirosis %	-	-	-	-		6.0
Drowning %	-	-	-	3.0	-	
Motor vehicle accident	-	-	1.5	-	-	-
Comorbid conditions		-		-	-	-
Diabetes mellitus %	75	57	74	75	38	54
Chronic renal disease %	11	9.7	9.7	19	10	6.0
Tuberculosis %	-	-	-	-	16	9.0
Immune disorders/steroid therapy %	9.5	6.2	2.9	3.0	4.0	6.0
Solid tumors %	4.4	-	0.7	-	10	1.5
Hematological malignancies %	-	-		0.7	8.0	
Chronic lung disease %	-	2.8	3.0	-	-	-
Chronic heart disease %	-	-	-	-	-	7.0
Smoking %	-	-	-	-	-	10
Chronic alcoholism %	-	-	-	0.7	2.0	3.0
Hemolytic anemia %	-	-	-	0.7	2.0	
Malnutrition/anemia %	-	-	-	-	8.0	-

* Derived; % calculated as percentage of total number of cases; - Not reported; ^†^ Computed as a % of those with known race.

**Table 2:** Clinical manifestations from previously published case series or reports from Malaysia.

	Laboratory or Registry Data					Case Reports
	Zueter et al. [31] (*n* = 158)	Hassan et al. [32] (*n* = 145)	How et al. [36] (*n* = 135)	Pagalavan [33] (*n* = 44)	Puthucheary et al. [18] (*n* = 50)	Kingsley et al. [37] (*n* = 67)
Clinical presentations						
Acute pulmonary %	41	-	41	63	58	33
Acute blood stream %	-	-	19	13	24	61
Disseminated %	29	-	16	-	30	37
Localized %	-	-	-	-	10	9.0
Primary diagnostic groups						
Pulmonary %	41	42	41	56	58	36
Soft tissue abscess/skin %	28	17	-	19	24	36
Bone and joint %	13	4.8	-	6.3	12	6.0
Genitourinary %	3.2	-	-	-	10	7.5
Neurologic %	5.7	4.8	-	-	6.0	7.5
No clinical focus %	22	-	19	13	24	7.5
Primary or secondary foci						
Liver abscess %	12	8.3	3.0	4.5	4.0	18
Splenic abscess %	9.5	10	3.0	9.1	2.0	12
Prostate abscess % ^†^	2.6	0.9	-	-	-	13
Parotid abscess %	2.5	-	-	-	-	1.5
Mycotic pseudoaneurysm %	-	-	-	-	-	7.5
Heart valve vegetation %	-	-	-	3.0	-	-
Pericardial effusion %	-	-	-	-	2.0	1.0
Bacteraemia %	77	52	94	59	100	61
Septic shock %	34	-	-	-	16	19

% Calculated as percentage of total number of cases; - Not reported; ^†^ Computed for males.

**Table 3:** Comparison of results from the four largest studies describing melioidosis among children in Malaysia.

	How et al. [46]	Sam et al. [47]	Fong et al. [44]	Mohan et al. [35]
Geographic area	Pahang	Kuala Lumpur	Sabah	Sarawak
Time period	2000–2003	1976–2005	2001–2012	2009–2014
Inclusion criteria	Culture-confirmed, age < 18 years	Culture-confirmed, age < 15 years	Culture-confirmed, age < 15 years	Culture-confirmed, age < 15 years
Number of cases	13	16	27	42
Annual incidence per 100,000 children	0.7	-	0.6	4.1
Age, median (years)	9.5 *	9.7 *	7.0	4.7
Male/female ratio	3.3:1	4.3:1	1.3:1	1.0:1
Underlying medical conditions (%)	0	69	52	0
Localized disease (%)	46	56	7 ^‡^	45
Bacteraemia (%)	54	44	74	48
Septicaemic shock (%)	38	-	52	31
Fatality rate (%)	31	33 ^†^	59	24

* Mean; ^†^ Includes one child who was taken home in an extremely ill state having failed to respond to ceftazidime, and is presumed to have died; ^‡^ Includes one child who had liver/splenic abscesses but no other focus of infection or bacteraemia.

**Table 4:** Mortality and culture-confirmed recurrence from previously published case series or reports from Malaysia.

	Laboratory or Registry Data					Case Reports
	Zueter et al. [31] (*n* = 158)	Hassan et al. [32] (*n* = 145)	How et al. [36] (*n* = 135)	Pagalavan [33] (*n* = 44)	Puthucheary et al. [18] (*n* = 50)	Kingsley et al. [37] (*n* = 67)
Mortality %	33	34	54	48	65	43
Bacteraemic %	-	48	59	-	65	59
Nonbacteraemic %	-	19	-	-	-	0.0
^1^ Recurrence %	2.6	-	19	-	4.0	9.0

% Calculated as percentage of total number of cases; - Not reported; ^1^ Recurrent is defined as melioidosis infection following the completion of their antibiotic therapy, which may be culture-confirmed or based on clinical presentation.

## References

[1] Stanton A.T., Fletcher W. (1925). Melioidosis: A disease of rodents communicable to man. Lancet.

[2] Stanton A.T., Flectcher W., Kanagarayer K. (1924). Two cases of melioidosis. J. Hyg. (London).

[3] Thin R.N.T., Brown M., Stewart J.B., Garrett C.J. (1970). Melioidosis: A report of ten cases. QJM Int. J. Med..

[4] Strauss J.M., Jason S., Mariappan M. (1967). *Pseudomonas pseudomallei* in soil and surface water of Sabah, Malaysia. Med. J. Malays..

[5] Strauss J.M., Alexander A.D., Rapmund G., Gan E., Dorsey A.E. (1969). Melioidosis in Malaysia: III. Antibodies to *Pseudomonas pseudomallei* in the human population. Am. J. Trop. Med. Hyg..

[6] Strauss J., Ellison D., Gan E., Jason S., Marcarelli J.L., Rapmund G. (1969). Melioidosis in Malaysia. IV. Intensive ecological study of Carey Island, Selangor, for *Pseudomonas pseudomallei*. Med. J. Malays..

[7] Strauss J.M., Groves M.G., Mariappan M., Ellison D.W. (1969). Melioidosis in Malaysia. II. Distribution of *Pseudomonas pseudomallei* in soil and surface water. Am. J. Trop. Med. Hyg..

[8] Stanton A.T., Fletcher W. (1932). Melioidosis.

[9] Mustaffa Babjee A., Nor Aidah A.R., Puthucheary S.D., Malik Y.A. (1994). Melioidosis in animals. Melioidosis: Prevailing Problems and Future Directions.

[10] Vellayan S., Puthucheary S.D., Malik Y.A. (1994). Melioidosis in zoo animals in Malaysia. Melioidosis: Prevailing Problems and Future Directions.

[11] Puthucheary S.D. (2009). Melioidosis in Malaysia. Med. J. Malays..

[12] Naama T., Norazura A.H., Chin S.W., Mazlan L., Nurul Fatiha A.S., Masrin A., Naheed M.H., Ramlan M. Melioidosis in various animal species diagnosed in the Veterinary Research Institute from 2007 to 2011. Proceedings of the International Conference on One Health and 24th VAM Congress.

[13] Lim M.L., Ismail S.S., Rahman N., Watanabe M. (2015). Melioidosis: A localised osteomyelitis in a cat. J. Vet. Malaya.

[14] De Silva G.S. (1971). Notes on the orang-utan rehabilitation project in Sabah. Malays. Nat. J..

[15] Idris A., Rachmat R.F.N., Ali S.M.M. (1998). Melioidosis: A case of sheep to human transmission. J. Vet. Malays..

[16] Puthucheary S.D., Lin H.P., Yap P.K. (1981). Acute septicaemic melioidosis: A report of seven cases. Trop. Geogr. Med..

[17] Yee K.C., Lee M.K., Chua C.T., Puthucheary S.D. (1988). Melioidosis, the great mimicker: A report of 10 cases from Malaysia. J. Trop. Med. Hyg..

[18] Puthucheary S.D., Parasakthi N., Lee M.K. (1992). Septicaemic melioidosis: A review of 50 cases from Malaysia. Trans. R. Soc. Trop. Med. Hyg..

[19] Noordin K., Abdullah M.M., Natarjan C., Wahab Y.A., Abdullah K. (1995). Pseudoaneurysm of the renal artery associated with melioidosis. Br. J. Urol..

[20] Nathan S.A., Puthucheary S.D. (2005). An electronmicroscopic study of the interaction of *Burkholderia pseudomallei* and human macrophages. Malays. J. Pathol..

[21] Francis A., Aiyar S., Yean C.Y., Naing L., Ravichandran M. (2006). An improved selective and differential medium for the isolation of *Burkholderia pseudomallei* from clinical specimens. Diagn. Microbiol. Infect. Dis..

[22] Su Y.C., Wan K.L., Mohamed R., Nathan S. (2008). A genome level survey of *Burkholderia pseudomallei* immunome expressed during human infection. Microbes Infect..

[23] Chua K.H., See K.H., Thong K.L., Puthucheary S.D. (2010). DNA fingerprinting of human isolates of *Burkholderia pseudomallei* from different geographical regions of Malaysia. Trop. Biomed..

[24] Puthucheary S.D., Puah S.M., Chai H.C., Thong K.L., Chua K.H. (2012). Molecular investigation of virulence determinants between a virulent clinical strain and an attenuated strain of *Burkholderia pseudomallei*. J. Mol. Microbiol. Biotechnol..

[25] Wong Y.C., Pain A., Nathan S. High-throughput sequencing of large-scale transposon mutants: A genetic tool to identify essential genes of *Burkholderia pseudomallei*. Proceedings of the 7th World Melioidosis Congress.

[26] Podin Y., Sarovich D.S., Price E.P., Kaestli M., Mayo M., Hii K., HieUng N., Wong S., Wong I., Wong J. (2014). *Burkholderia pseudomallei* isolates from Sarawak, Malaysian Borneo, are predominantly susceptible to aminoglycosides and macrolides. Antimicrob. Agents Chemother..

[27] Vellasamy K.M., Mariappan V., Shankar E.M., Vadivelu J. (2016). *Burkholderia pseudomallei* differentially regulates host innate immune response genes for intracellular survival in lung epithelial cells. PLoS Negl. Trop. Dis..

[28] Mariappan V., Vellasamy K.M., Vadivelu J. (2017). Host-adaptation of *Burkholderia pseudomallei* alters metabolism and virulence: A global proteome analysis. Sci. Rep..

[29] Currie B.J., Fisher D.A., Howard D.M., Burrow J.N., Lo D., Selva-Nayagam S., Anstey N.M., Huffam S.E., Snelling P.L., Marks P.J. (2000). Endemic melioidosis in tropical northern Australia: A 10-year prospective study and review of the literature. Clin. Infect. Dis..

[30] Cheng A., Currie B. (2005). Melioidosis: Epidemiology, pathophysiology, and management. Clin. Microbiol. Rev..

[31] Zueter A.R., Yean C.Y., Abumarzouq M., Rahman Z.A., Deris Z.Z., Harun A. (2016). The epidemiology and clinical spectrum of melioidosis in a teaching hospital in a north-eastern state of Malaysia: A fifteen-year review. BMC Infect. Dis..

[32] Hassan M.R.A., Pani S.P., Peng N.P., Voralu K., Vijayalakshmi N., Mehanderkar R., Aziz N.A., Michael E. (2010). Incidence, risk factors and clinical epidemiology of melioidosis: A complex socio-ecological emerging infectious disease in the Alor Setar region of Kedah, Malaysia. BMC Infect. Dis..

[33] Pagalavan L. (2005). Melioidosis: The Johor Bahru experience. Med. J. Malays..

[34] Melioidosis—Databases. http://www.melioidosis.info/info.aspx?pageID=107.

[35] Mohan A., Podin Y., Tai N., Chieng C.-H., Rigas V., Machunter B., Mayo M., Wong D., Chien S.-L., Tan L.-S. (2017). Pediatric melioidosis in Sarawak, Malaysia: Epidemiological, clinical and microbiological characteristics. PLoS Negl. Trop. Dis..

[36] How S.H., Ng K.H., Jamalludin A.R., Shah A., Rathor Y. (2005). Melioidosis in Pahang, Malaysia. Med. J. Malays..

[37] Kingsley P.V., Leader M., Nagodawithana N.S., Tipre M., Sathiakumar N. (2016). Melioidosis in Malaysia: A review of case reports. PLoS Negl. Trop. Dis..

[38] Chandni R. (2013). Melioidosis: The great mimicker. Medicine Update.

[39] Pruekprasert P., Jitsurong S. (1991). Case report: Septicemic melioidosis following near drowning. Southeast Asian J. Trop. Med. Public Health.

[40] Sapian M., Khairi M.T., How S.H., Rajalingam R., Sahhir K., Norazah A., Khebir V., Jamalludin A.R. (2012). Outbreak of melioidosis and leptospirosis co-infection following a rescue operation. Med. J. Malays..

[41] Currie B.J., Ward L., Cheng A.C. (2010). The epidemiology and clinical spectrum of melioidosis: 540 cases from the 20-year Darwin prospective study. PLoS Negl. Trop. Dis..

[42] Limmathurotsakul D., Wongratanacheewin S., Teerawattanasook N., Wongsuvan G., Chaisuksant S., Chetchotisakd P., Chaowagul W., Day N.P.J., Peacock S.J. (2010). Increasing incidence of human melioidosis in northeast Thailand. Am. J. Trop. Med. Hyg..

[43] Kingsley P.V., Arunkumar G., Tipre M., Leader M., Sathiakumar N. (2016). Pitfalls and optimal approaches to diagnose melioidosis. Asian Pac. J. Trop. Med..

[44] Fong S.M., Wong K.J., Fukushima M., Yeo T.W. (2015). Thalassemia major is a major risk factor for pediatric melioidosis in Kota Kinabalu, Sabah, Malaysia. Clin. Infect. Dis..

[45] Sanderson C., Currie B.J. (2014). Melioidosis: A pediatric disease. Pediatr. Infect. Dis. J..

[46] How H.S., Ng K.H., Yeo H.B., Tee H.P., Shah A. (2005). Pediatric melioidosis in Pahang, Malaysia. J. Microbiol. Immunol. Infect..

[47] Sam I.C., Puthucheary S.D. (2006). Melioidosis in children from Kuala Lumpur, Malaysia. Ann. Trop. Paediatr..

[48] McLeod C., Morris P.S., Bauert P.A., Kilburn C.J., Ward L.M., Baird R.W., Currie B.J. (2015). Clinical presentation and medical management of melioidosis in children: A 24-year prospective study in the Northern Territory of Australia and review of the literature. Clin. Infect. Dis..

[49] Turner P., Kloprogge S., Miliya T., Soeng S., Tan P., Sar P., Yos P., Moore C.E., Wuthiekanun V., Limmathurotsakul D. (2016). A retrospective analysis of melioidosis in Cambodian children, 2009–2013. BMC Infect. Dis..

[50] Thatrimontrichai A., Maneenil G. (2012). Neonatal melioidosis: Systematic review of the literature. Pediatr. Infect. Dis. J..

[51] Lumbiganon P., Viengnondha S. (1995). Clinical manifestations of melioidosis in children. Pediatr. Infect. Dis. J..

[52] Ashdown L.R. (1979). An improved screening technique for isolation of *Pseudomonas pseudomallei* from clinical specimens. Pathology.

[53] Wiersinga W.J., Currie B.J., Peacock S.J. (2012). Melioidosis. N. Engl. J. Med..

[54] Podin Y., Kaestli M., McMahon N., Hennessy J., Ngian H.U., Wong J.S., Mohana A., Wong S.C., William T., Mayo M. (2013). Reliability of automated biochemical identification of *Burkholderia pseudomallei* is regionally dependent. J. Clin. Microbiol..

[55] Hoffmaster A.R., Aucoin D., Baccam P., Baggett H.C., Baird R., Bhengsri S., Blaney D.D., Brett P.J., Brooks T.J. G., Brown K.A. (2015). Melioidosis diagnostic workshop, 2013. Emerg. Infect. Dis..

[56] Trinh T.T., Hoang T.S., Tran D.A., Trinh V.T., Göhler A., Nguyen T.T., Hoang S.N., Krumkamp R., Nguyen L.T.N., May J. (2017). A simple laboratory algorithm for diagnosis of melioidosis in resource-constrained areas: A study from north-central Vietnam. Clin. Microbiol. Infect..

[57] Mohd Noor A., Ahmad N., Rozita W., Mahiyuddin W. (2015). The optimization of IgM in-house ELISA for the laboratory diagnosis of melioidosis in Malaysia. Int. J. Pathol. Clin. Res..

[58] Novak R.T., Glass M.B., Gee J.E., Gal D., Mayo M.J., Currie B.J., Wilkins P.P. (2006). Development and evaluation of a real-time PCR assay targeting the type III secretion system of *Burkholderia pseudomallei*. J. Clin. Microbiol..

[59] Richardson L.J., Kaestli M., Mayo M., Bowers J.R., Tuanyok A., Schupp J., Engelthaler D., Wagner D.M., Keim P.S., Currie B.J. (2012). Towards a rapid molecular diagnostic for melioidosis: Comparison of DNA extraction methods from clinical specimens. J. Microbiol. Methods.

[60] Deris Z.Z., Hasan H., Suraiya M.N.S. (2010). Clinical characteristics and outcomes of bacteraemic melioidosis in a teaching hospital in a northeastern state of Malaysia: A five-year review. J. Infect. Dev. Ctries..

[61] Chaowagul W., White N.J., Dance D.A.B., Wattanagoon Y., Naigowit P., Davis T.M.E., Looareesuwan S., Pitakwatchara N. (1989). Melioidosis: A major cause of community-acquired septicemia in northeastern Thailand. J. Infect. Dis..

[62] Lim K.S., Chong V.H. (2010). Radiological manifestations of melioidosis. Clin. Radiol..

[63] Khosravi Y., Vellasamy K.M., Mariappan V., Ng S.-L., Vadivelu J. (2014). Antimicrobial susceptibility and genetic characterisation of *Burkholderia pseudomallei* isolated from Malaysian patients. Sci. World J..

[64] Zueter A.R., Rahman Z.A., Abumarzouq M., Harun A. (2018). Multilocus sequence types of clinical *Burkholderia pseudomallei* isolates from peninsular Malaysia and their associations with disease outcomes. BMC Infect. Dis..

[65] Godoy D., Randle G., Simpson A.J., Aanensen D.M., Pitt T.L., Kinoshita R., Spratt B.G. (2003). Multilocus sequence typing and evolutionary relationships among the causative agents of melioidosis and glanders, *Burkholderia pseudomallei* and *Burkholderia mallei*. J. Clin. Microbiol..

[66] McCombie R.L., Finkelstein R.A., Woods D.E. (2006). Multilocus sequence typing of historical *Burkholderia pseudomallei* isolates collected in Southeast Asia from 1964 to 1967 provides insight into the epidemiology of melioidosis. J. Clin. Microbiol..

[67] Cheng A.C., Godoy D., Mayo M., Gal D., Spratt B.G., Currie B.J. (2004). Isolates of *Burkholderia pseudomallei* from northern Australia are distinct by multilocus sequence typing, but strain types do not correlate with clinical presentation. J. Clin. Microbiol..

[68] Cheng A.C., Day N.P.J., Mayo M.J., Gal D., Currie B.J. (2005). *Burkholderia pseudomallei* strain type, based on pulsed-field gel electrophoresis, does not determine disease presentation in melioidosis. Microbes Infect..

[69] Chin C.Y., Monack D.M., Nathan S. (2010). Genome wide transcriptome profiling of a murine acute melioidosis model reveals new insights into how *Burkholderia pseudomallei* overcomes host innate immunity. BMC Genom..

[70] Chin C.Y., Monack D.M., Nathan S. (2012). Delayed activation of host innate immune pathways in streptozotocin-induced diabetic hosts leads to more severe disease during infection with *Burkholderia pseudomallei*. Immunology.

[71] Lee S.H., Ooi S.K., Mahadi N.M., Tan M.W., Nathan S. (2011). Complete killing of *Caenorhabditis elegans* by *Burkholderia pseudomallei* is dependent on prolonged direct association with the viable pathogen. PLoS ONE.

[72] Ooi S.K., Lim T.Y., Lee S.H., Nathan S. (2012). *Burkholderia pseudomallei* kills *Caenorhabditis elegans* through virulence mechanisms distinct from intestinal lumen colonization. Virulence.

[73] Lee S.H., Wong R.R., Chin C.Y., Lim T.Y., Eng S.A., Kong C., Ijap N.A., Lau M.S., Lim M.P., Gan Y.H. (2013). *Burkholderia pseudomallei* suppresses *Caenorhabditis elegans* immunity by specific degradation of a GATA transcription factor. Proc. Natl. Acad. Sci. USA.

[74] Chieng S., Carreto L., Nathan S. (2012). *Burkholderia pseudomallei* transcriptional adaptation in macrophages. BMC Genom..

[75] Lee S.H., Chong C.E., Lim B.S., Chai S.J., Sam K.K., Mohamed R., Nathan S. (2007). *Burkholderia pseudomallei* animal and human isolates from Malaysia exhibit different phenotypic characteristics. Diagn. Microbiol. Infect. Dis..

[76] Liew S.M., Tay S.T., Wongratanacheewin S., Puthucheary S.D. (2012). Enzymatic profiling of clinical and environmental isolates of *Burkholderia pseudomallei*. Trop. Biomed..

[77] Vellasamy K.M., Mariappan V., Hashim O., Vadivelu J. (2012). *Burkholderia pseudomallei* host-pathogen interactions: Role of live bacteria and secretory proteins. Int. J. Infect. Dis..

[78] Kang W.T., Vellasamy K.M., Vadivelu J. (2016). Eukaryotic pathways targeted by the type III secretion system effector protein, BipC, involved in the intracellular lifecycle of *Burkholderia pseudomallei*. Sci. Rep..

[79] Kang W.T., Vellasamy K.M., Rajamani L., Beuerman R.W., Vadivelu J. (2016). *Burkholderia pseudomallei* type III secreted protein BipC: Role in actin modulation and translocation activities required for the bacterial intracellular lifecycle. PeerJ.

[80] Vadivelu J., Vellasamy K.M., Thimma J., Mariappan V., Kang W.T., Choh L.C., Shankar E.M., Wong K.T. (2017). Survival and intra-nuclear trafficking of *Burkholderia pseudomallei*: Strategies of evasion from immune surveillance?. PLoS Negl. Trop. Dis..

[81] Ramli N.S.K., Eng Guan C., Nathan S., Vadivelu J. (2012). The effect of environmental conditions on biofilm formation of *Burkholderia pseudomallei* clinical isolates. PLoS ONE.

[82] Chin C.Y., Hara Y., Ghazali A.K., Yap S.J., Kong C., Wong Y.C., Rozali N., Koh S.F., Hoh C.C., Puthucheary S.D. (2015). Global transcriptional analysis of *Burkholderia pseudomallei* high and low biofilm producers reveals insights into biofilm production and virulence. BMC Genom..

[83] Yew K.L. (2013). Antimicrobial prophylaxis for melioidosis and leptospirosis for at risk rescue workers. Med. J. Malays..

[84] How S.H., Ng T.H., Jamalludin A.R., Tee H.P., Kuan Y.C., Alex F., Aminudin C.A., Sapari S., Quazi M.H. (2009). Pahang melioidosis registry. Med. J. Malays..

[85] Suleiman M., Flecia K., Ponolin P., Jasni G. (2014). Guideline for Clinical and Public Health Management of Melioidosis in Sabah.

